# Fabrication and Performance Evaluation of a Nanostructured ZnO-Based Solid-State Electrochromic Device

**DOI:** 10.1021/acsami.4c10545

**Published:** 2024-09-16

**Authors:** Marivone Gusatti, Daniel Aragão Ribeiro de Souza, Mario Barozzi, Rossana Dell’Anna, Elena Missale, Lia Vanzetti, Massimo Bersani, Marcelo Nalin

**Affiliations:** † Institute of Chemistry, Department of Analytical, Physical, and Inorganic Chemistry, São Paulo State University (UNESP), Araraquara 14800-060, São Paulo, Brazil; ‡ Sensors and Devices Center, Bruno Kessler Foundation (FBK), via Sommarive, 18, Povo, Trento 38123, Trentino, Italy

**Keywords:** PEDOT:PSS, Al counter electrode, ZnO-based ECD, coloration efficiency, electrochromic response, spray coating technique

## Abstract

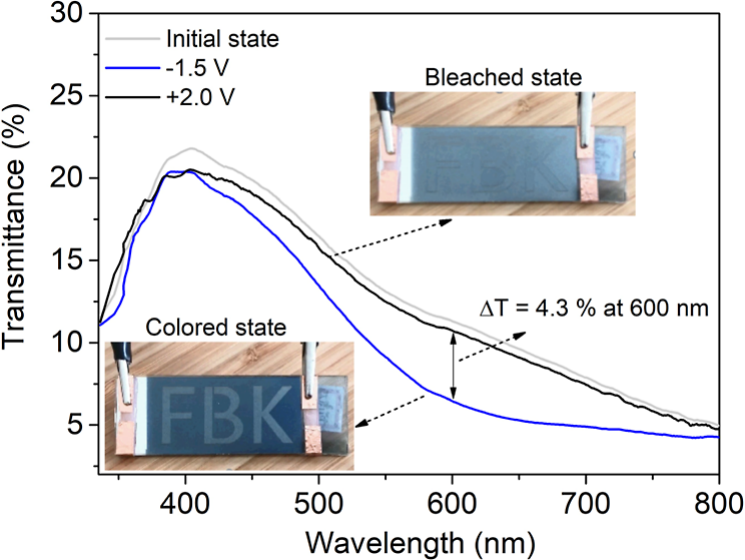

In this study, we present an all-solid-state electrochromic device (ECD) that eliminates the need for hard-to-obtain materials and conventional liquid/gel electrolytes. Using a cost-effective and industrially scalable spray coating technique, we developed an ECD containing a layer of zinc oxide nanorods (ZnO_nano_) synthesized via a simple solochemical route. The device configuration includes a preformed Al-coated glass substrate, acting as a counter electrode, within a glass/Al/ZnO_nano_/PEDOT:PSS architecture. The device exhibits reversible switching between light blue and dark blue states upon application of −1.2 V and +2.8 V, respectively, with a significant difference in transmittance between bleached and colored states in the visible-NIR spectrum, featuring a high coloration efficiency of 275.62 cm^2^/C at 600 nm. The response times required for both coloring and bleaching states were 9.92 s and 7.51 s, respectively, for a sample with an active area of 5.5 × 2.5 cm^2^. Regarding the electrochemical stability of the ZnO-based ECD, the transmittance modulation reached around 8.01% at 600 nm after 12,800 s, following initial variations observed during the first 10 cycles. These results represent significant progress in electrochromic technology, offering a sustainable and efficient alternative to traditional ECDs. The use of economical fabrication techniques and the exclusion of critical materials highlight the potential for widespread industrial adoption of this novel ECD design.

## 1 Introduction

Given the increasing challenges related to sustainability and energy efficiency, electrochromic (EC) devices are considered a promising solution for dynamic coloration, with significant potential across various applications.^1−3^ Electrochromism is a phenomenon characterized by reversible color changes in certain materials induced by electrochemical redox reactions.^4−7^ This color change typically occurs between a colored (darkened) state and transmissive (bleached) state or between two distinct colored states.^5,6^ Electrochromic devices (ECDs) provide a unique capability to dynamically control light transmission, offering opportunities for maximizing the utilization of natural light.^8^ This feature makes them especially advantageous for applications in energy-saving devices such as smart windows,^9−11^ which can dynamically modulate optical properties to enhance indoor comfort, thereby reducing energy consumption for lighting and cooling in both residential and commercial buildings.^12,13^

Despite their promise, ECDs face significant challenges related to certain materials, durability, and manufacturing processes.^14^ These devices traditionally rely on critical components such as indium tin oxide (ITO) and tungsten trioxide (WO_3_), which are not only costly and scarce but also environmentally unfriendly.^15−19^ Additionally, the use of liquid and gel electrolytes, common in conventional ECD structures, presents concerns regarding leakage, low chemical stability, and internal short-circuit issues.^20−23^ Moreover, many of the manufacturing processes that involve these critical materials are complex and expensive, further complicating large-scale ECD production.^24^ Addressing these interconnected challenges represents a crucial step toward enabling the widespread adoption of ECD technology.

Recent advancements in electrochromic technology have led to the emergence of solid-state EC devices, aimed at overcoming limitations associated with traditional liquid or gel electrolyte-based systems.^25,26^ Solid-state ECDs offer enhanced robustness and durability, providing a more reliable solution for various applications. Furthermore, solid-state EC devices, achieved through layer-by-layer processing in a single direction using cost-effective techniques, not only advance EC technology but also facilitate scalability for large-area ECD production and market adoption.^27−29^

In this context, we present a simplified all-solid-state device assembled with only two sequential layers in one direction, essentially in a continuous process, on a single glass substrate coated with the counter electrode (CE) material. Leveraging the unique properties of zinc oxide (ZnO) nanorods (ZnO_nano_), synthesized in this work through a simple solochemical method, our ECD eliminates the need for ITO, tungsten oxide, lithium, and any other liquid/gel electrolytes.

Our ZnO-based ECD was fabricated by depositing a poly(3,4-ethylenedioxythiophene): polystyrenesulfonate (PEDOT:PSS) solution on a ZnO_nano_ film previously sprayed on a preformed Al-coated glass substrate. Both PEDOT:PSS and ZnO_nano_ films were applied using a simple spray coating process. This technique notably simplifies ECD fabrication, effectively reducing production costs and the waste of raw materials. In addition, by incorporating PEDOT:PSS as both the working electrode (WE) and electrochromic material, we have further simplified our fabrication technology, reducing the number of device layers and minimizing risks associated with material incompatibility and short circuits. Furthermore, to the best of our knowledge, this represents the first development of an all-solid-state ECD incorporating ZnO nanostructures as an intermediate layer between the WE and the Al counter electrode. Thus, our approach not only has the potential to facilitate scalable production of ECDs, thereby increasing manufacturing speed, but also advances the field of electrochromic technology, paving the way for solid-state devices with enhanced performance and long-term stability.

Raman spectroscopy, secondary ion mass spectrometry (SIMS), scanning electron microscopy (SEM), and profilometry techniques were employed to assess the elemental composition, morphology, and surface characteristics of the Al and PEDOT:PSS films deposited on glass substrates, as well as the ECD before and after 167 cycles of consecutive switching. Additionally, the optical and electrochemical properties of the glass/Al/ZnO_nano_/PEDOT:PSS device were investigated in detail. The ZnO-based ECD with an all-solid-state configuration exhibited two distinct color states corresponding to variations in applied potentials, demonstrating good optical contrast, high coloration efficiency, and relatively fast response times. These attributes make the device promising for applications in security features and displays technology.

## 2 Materials and Methods

### 2.1 Materials

The primary materials utilized in the experiments were: poly(3,4-ethylenedioxythiophene): poly(styrenesulfonate) (PEDOT:PSS, 99.5% purity), diethylene glycol (DEG, C_4_H_10_O_3_, 99% purity), aluminum pellets (Al, 99.999% purity), acetone (Ac, CH_3_COCH_3_, 99.9% purity), ethanol (C_2_H_5_OH, 99.8% purity), methanol (MeOH, CH_3_OH, 99.9% purity), isopropyl alcohol (IPA, C_3_H_8_O, 99.9% purity), sodium hydroxide (NaOH, 99.5% purity), and zinc nitrate hexahydrate (Zn(NO_3_)_2_·6H_2_O, 99.9% purity). All reagents were purchased from Sigma-Aldrich and were used without additional purification.

### 2.2 Methods

#### 2.2.1 Solochemical Synthesis of ZnO Nanostructures

**Figure 1:**
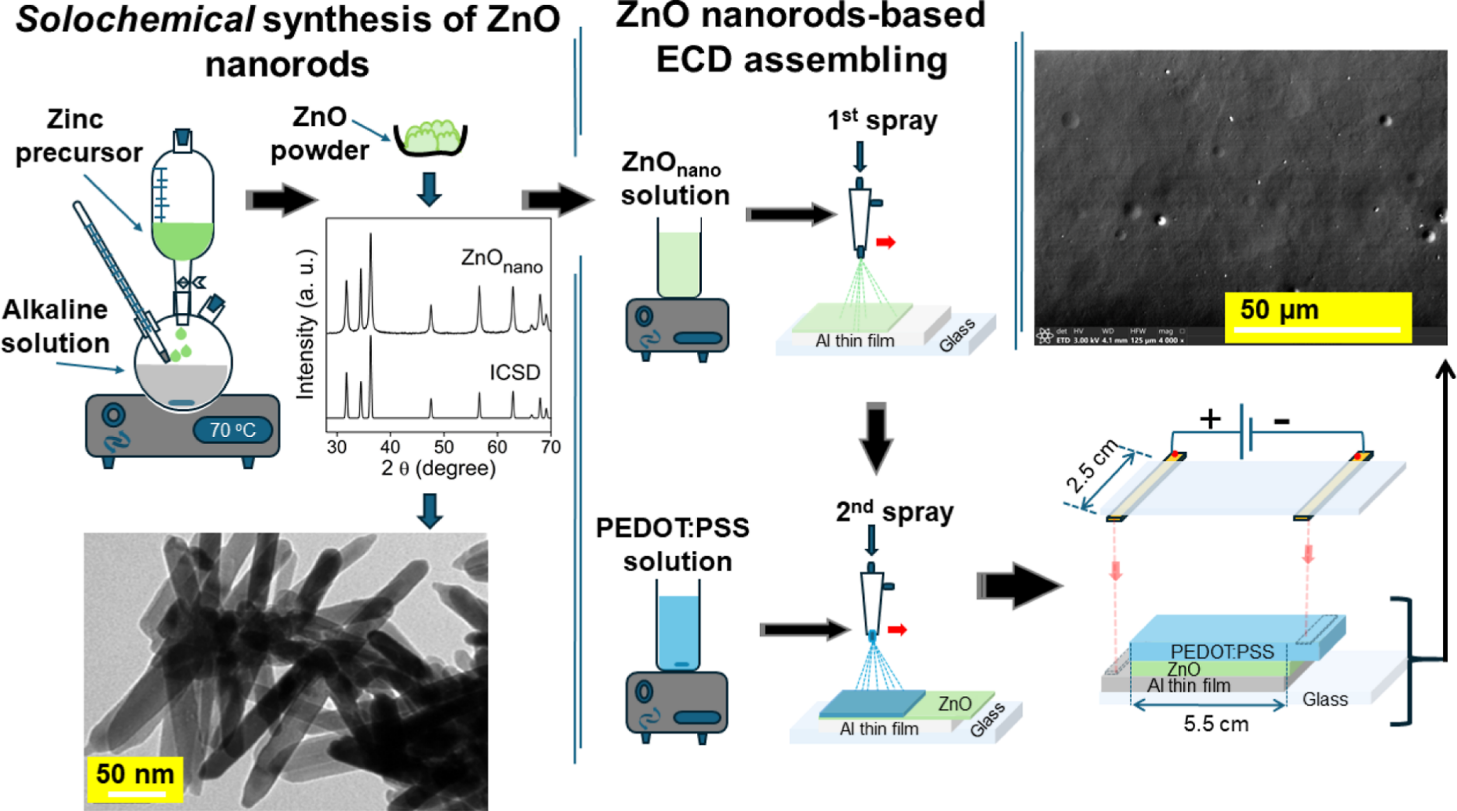
Schematic illustration of the procedure employed to prepare the ZnO nanorods used as the intermediate layer between the WE and CE, and the assembly of the electrochromic device, including X-ray diffraction (XRD) and transmission electron microscopy (TEM) of ZnO nanorods, and SEM of the ECD.

This study follows an experimental procedure similar to that outlined in our previous publications^30,31^ for the ECD fabrication, incorporating a substantial modification in the components constituting the layers of the electrochromic device. Specifically, the main change involves replacing tris(8-hydroxyquinoline) aluminum(III) (Alq_3_) with nanostructured ZnO synthesized in this work. The synthesis of ZnO nanostructures followed the methodology detailed in our previous publications,^32,33^ employing an environmentally friendly solochemical route at a relatively low reaction temperature. In this synthesis process, a 1 M solution of NaOH was prepared by dissolving NaOH in deionized water, followed by agitation and heating until reaching a temperature of 70 °C. Subsequently, a 0.5 M solution of zinc nitrate hexahydrate (dissolved in deionized water) was added dropwise to the NaOH solution over 1 h. The resulting mixture was stirred at 70 °C for 3 h in a reflux system. After the complete reaction, the material was filtered and subsequently dried at 65 °C to yield the ZnO powder (Figure 1). This resultant sample was then utilized to prepare a solution, which was subsequently deposited onto an Al-coated glass substrate during the assembly of the electrochromic device.

Thus, the solochemical method offers a simple and cost-effective approach for synthesizing ZnO nanostructures. Integrating this material, synthesized through this technique, into the production of ECDs not only reduces manufacturing costs for traditional electrochromic devices but also presents a cost-saving opportunity compared to our previous device,^31^ where Alq_3_ was used as a buffer material layer.

#### 2.2.2 Substrate Preparation

For aluminum (Al) deposition, microscope glass slides were selected as the substrates for the devices. These substrates underwent sequential cleaning processes, including ultrasonication for 10 min with detergent, deionized water (DI), acetone, and ethanol, respectively, followed by drying with nitrogen gas. Subsequently, Al films were deposited on the glass substrates using the electron beam evaporation technique (Auto 500 EB3 Source System made by HHV Ltd., United Kingdom). The deposition process utilized an electron beam of 5 kV in a vacuum of 8 × 10^–6^ Pa to construct the counter electrode (CE) of the ECDs. The deposition rate for the thin films was approximately 12 nm/min, resulting in a thickness of about 36 nm. At this thickness, the Al film exhibited adequate conductivity, which is crucial since it will be used as the counter electrode in the ECD, playing an important role in ensuring proper electrochromic performance.

#### 2.2.3 Layers Deposition

In this study, the layers of our solid-state ZnO-based ECD were assembled using the spray coating technique. This process involved the sequential deposition of only two layers, starting from the Al-coated glass substrate to the top of the device, in an almost continuous process. Prior to deposition, solutions of ZnO nanostructures and PEDOT:PSS were meticulously prepared as follows: To prepare the ZnO_nano_ film, the as-synthesized ZnO_nano_ powder was dissolved in methanol at a concentration of 2.5 mg/mL. The resulting solution underwent ultrasonic stirring at room temperature for 30 min to obtain a homogeneous mixture suitable for deposition on the Al-coated glass substrate. Meanwhile, the pristine PEDOT:PSS solution was prepared using the same mixing ratio of PEDOT:PSS, DEG, and IPA employed in our previous research.^31^

The formulated ZnO_nano_ solution was directly sprayed on the Al-coated glass at 50 °C and dried in an oven at the same temperature for 60 min to ensure complete removal of solvent vapor. Then, the pristine PEDOT:PSS solution, which was used both as the electrochromic layer and the WE for the fabrication of our ECD, was sprayed on the ZnO_nano_ film at a temperature of 50 °C. The solutions were pumped with a syringe pump equipment (Cole Parmer Instrument Co., CAT no. 78-0202C, USA) through a spray nozzle. In the spray coater, the nozzle moves horizontally over the substrate, which is in contact with a hot plate. All other spray coating parameters for depositing the ZnO_nano_ and pristine solutions remained consistent with those described in our previous work^30^ for Alq_3_ and PEDOT:PSS solutions, respectively.

#### 2.2.4 ECD Assembling Procedure

Figure 1 presents a schematic representation of the electrochromic device assembly. Before depositing the Al, one edge of the glass slides was covered with a mask to create a clean area for the subsequent deposition of the WE. After the Al deposition, the edge of the glass substrate with Al was covered with a mask to prevent the deposition of other materials in that region, ensuring it serves as the counter electrode of the ECD. Consequently, excluding the areas designated for the working and counter electrodes, an electrochromic device with an active area of 5.5 × 2.5 cm^2^ can be fabricated.

The Al-coated substrate was then placed on a heating plate, with the nonconductive side of the glass in contact with the plate and the conducting side containing Al facing upward to initiate device assembly. At the selected temperature, the prepared ZnO_nano_ solution was sprayed onto the conductive side using a spray coating at a flow rate of 0.2 mL/min and a nozzle speed of 42 cm/min. This deposition was confined to the active area of the device. After depositing the ZnO_nano_, the coating was dried in a vacuum oven at 50 °C before applying the PEDOT:PSS layer. Next, the mask protecting the WE area was removed, and the pristine solution (used both as the EC material and WE) was sprayed onto the substrate at a flow rate of 0.1 mL/min, using the same spray coating speed as for the ZnO_nano_ solution. These deposition processes were repeated five times each for both the ZnO_nano_ and PEDOT:PSS films. Finally, the devices underwent a final drying process in a vacuum oven at 50 °C for 60 min.

After complete drying, another clean microscope glass substrate, of the same dimensions as that used for the device assembly, was carefully placed on top of the device. Copper tape was positioned along the edges of the clean substrate to establish electrical contact with the working and counter electrodes of the device. This configuration enabled electrical connections through probes. Both the clean substrate and device were then sealed together with adhesive tape to encapsulate the electrochromic device.

### 2.3 Characterization

The micro-Raman spectra were acquired using the LabRam HR Evolution (Horiba-Jobin-Yvon, France) confocal Raman spectrometer. All measurements were performed at room temperature, employing a 532 nm laser source with a spot diameter of 0.7 μm. The spectral range of 1100–1700 cm^–1^ was probed to characterize the samples with a grating of 300 L/mm. The spectra were compared after normalizing the area and subtracting the background.

The surface morphology was characterized using the Scanning Electron Microscope of a ThermoFisher Scientific Helios 5 PFIB/SEM instrument. For a comprehensive understanding of the layer microstructure beneath the surface, SIMS measurements were carried out with a dynamic SIMS Cameca SC-Ultra. A Cs^+^ primary ion beam with an impact energy of 1 keV and an incidence angle of 63° was used for surface sputtering, with a raster area of 250 × 250 μm^2^. Positive MCs^+^ molecular ions (where M represents each species of interest) were detected from a centered region of 75 × 75 μm^2^. All SIMS depth profiles were normalized point by point to the Cs^+^ secondary ion signal. The relative intensities (in counts per second) of the different secondary ion species do not represent their real relative concentrations, as these depend on the different ion yields for each matrix composition. The depth scale was calibrated by measuring the final sputtered craters with a Tencor P6 mechanical profilometer. To minimize depth uncertainty due to surface roughness, the whole stack thickness was measured after completely removing the active layers and exposing the flat surface of the glass substrate.

The electrochemical measurements were conducted at room temperature using a PGSTAT128N (METROHM Autolab B.V.) potentiostat/galvanostat analyzer in a three-electrode setup. The counter-electrode and WE were connected to the Al and PEDOT:PSS films, respectively, while the reference electrode was connected in series with the counter electrode. Cyclic voltammetry (CV) was performed with a scan rate of 100 mV s^–1^. Impedance spectra were measured at a voltage of 250 mV over a frequency range from 0.1 Hz to 1 MHz.

The UV–vis measurements were conducted using a UV–vis–NIR spectrophotometer (Cary 5000 Varian) connected to a potentiostat analyzer (METROHM μAutolabIII/FRA2), which was controlled by the NOVA software. In these analyses, the transmittance spectra were recorded using UV–vis software in “Scan mode”, with air used as the reference for baseline correction of the transmittance measurements. In contrast, in situ transmittance change recordings were performed using the “Kinetics mode” of the UV–vis software, where the initial state of the ECD was considered to be 100% transmittance at a specific wavelength. Thus, in situ transmittance measurements do not involve baseline correction and focus on the relative changes in transmittance (Δ*T*) as a function of applied potential over time.

During the UV–vis measurements, the ECD was inserted into the spectrometer, with its working and counter electrodes connected to the potentiostat. Transmittance changes were monitored relative to the applied potential. The procedure involved successive cycles, starting with the application of the coloration potential (−1.2 V) for 15 s, followed by linear sweep voltammetry (LSV) for 25 s, ranging from −1 V to 0 V. Subsequently, the bleaching potential (+2.8 V) was applied for 10 s, followed by another LSV for 25 s, ranging from +1 V to 0 V. These LSVs were recorded at a scan rate of 50 mV s^–1^. The described procedure was repeated for 167 cycles. The LSVs, conducted after each coloration and bleaching potential application, allowed for sample relaxation between consecutive cycles, enhancing the stability of the electrochromic behavior of the device.

Following the measurement, a mathematical adjustment was applied to the reference of 100% in situ transmittance change to ensure consistency over long cycle times with repeated potential switching. This mathematical correction was made solely to adjust the baseline of the switching data, which resulted in a baseline of 70% transmittance in the reduced state, without altering the relative transmittance difference (Δ*T*) between the reduced and oxidized states.

## 3 Results and Discussion

### 3.1 Structural and Morphological Characterization

The XRD patterns of the ZnO nanorods (ZnO_nano_) synthesized by solochemical processing at 70 °C, featuring a rod-like morphology with an average diameter of about 37 nm (Figure 1), show characteristic diffraction peaks corresponding to the hexagonal wurtzite structure of ZnO (space group *P*6_3_*mc*), consistent with ICSD Card no. 57450, as previously reported.^32,33^ No additional diffraction peaks indicative of other phases or impurities were detected in the XRD measurements, as can be seen in the XRD pattern included in Figure 1, confirming the successful synthesis of ZnO nanorods. Further characterization details regarding the synthesized ZnO can be found in the provided references. The use of nanostructured materials, particularly ZnO nanorods and nanowires, has emerged as a promising approach to further improve electrochromic performance. Such nanostructures can facilitate faster charge transport, reduce ion diffusion distances, and increase the number of active reaction sites for redox processes, thereby speeding up the ECD’s response.^34,35^ Given these advantages, the ZnO nanorods synthesized here using the practical and cost-effective solochemical method are expected to serve effectively as the intermediate layer between the WE and CE of the ECD. This configuration may enhance the efficiency of charge injection and extraction processes, potentially leading to improved performance and stability of the device.

**Figure 2:**
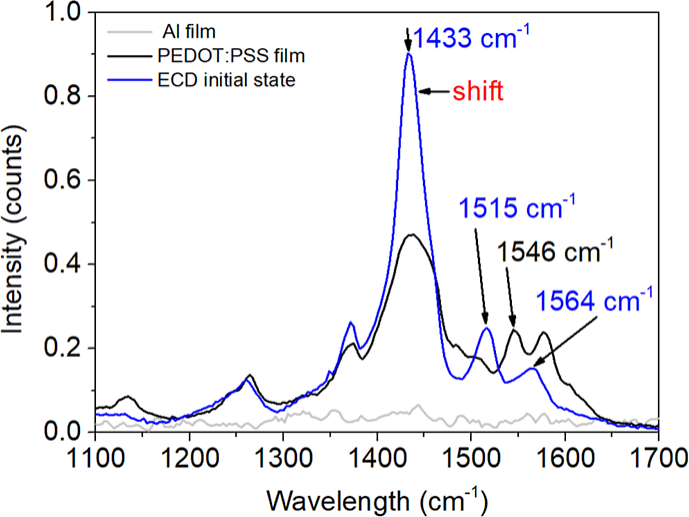
Raman spectra of the Al (gray line) and pristine PEDOT:PSS (black line) films deposited on glass substrates, along with the ECD in its initial state (blue line).

Figure 2 presents the Raman spectra of the PEDOT:PSS and Al films deposited on glass substrates, along with that of the as-fabricated ECD (initial state), which has not been tested or used previously. The spectrum of Al film (gray line) does not exhibit significant contributions. The spectrum of PEDOT:PSS film, to which DEG was added, exhibits typical features consistent with a PEDOT:PSS blend, according to the literature.^36−39^ It also reveals the PSS band at 1135 cm^–1^, attributed to sulfonic acid and sulfonate groups.^37,40^ A noteworthy observation is the presence of a discrete peak at 1546 cm^–1^. This peak likely indicates the formation of a composite between DEG and PEDOT:PSS, resulting in changes to the PEDOT:PSS chain conformation that could impact its electrical and optical properties.^41−43^

In turn, the Raman spectra of the ECD in its initial state (the line and peak positions are highlighted in blue) show notable absorption bands within the spectral range of 1200 and 1700 cm^–1^. These peaks, while slightly shifted, correspond closely to the characteristic vibrations reported for PEDOT in the literature.^39,40,44,45^ Specifically, the peak at 1260 cm^–1^ in the ECD, shifted from 1263 cm^–1^ in our PEDOT:PSS film, can be associated with the C_α_–C_α’_ inter-ring stretching vibration. Similarly, the peak at 1370 cm^–1^ in the ECD, close to the 1372 cm^–1^ position in our pristine film, corresponds to the C_β_–C_β_ stretching vibration. The most intense peak at 1433 cm^–1^ in the ECD, slightly shifted from 1436 cm^–1^ in our PEDOT:PSS film, can be assigned to the C_α_=C_β_ symmetric stretching vibration. The new peak at 1515 cm^–1^ (probably associated with thiophene rings in the middle of PEDOT chains), not present in our pristine PEDOT:PSS film, and the shifted peak at 1564 cm^–1^ (from 1577 cm^–1^ in our PEDOT:PSS film), related to thiophene rings at the end of PEDOT chains, both in the ECD, are likely related to the C_α_=C_β_ asymmetric stretching vibrations.

Notable differences in the Raman spectrum of the ECD, as highlighted in Figure 2, include some key features when comparing it to the Raman spectrum of the pristine film. The peak centered at 1433 cm^–1^ in the ECD exhibits slight broadening and increased intensity compared to the pristine film. Additionally, the ECD spectrum reveals a new peak at 1515 cm^–1^, which is absent in the PEDOT:PSS film. Another prominent feature is the peak at 1564 cm^–1^ in the ECD, which has shifted slightly from 1577 cm^–1^ in the pristine film. These differences also include a slight shift of the peaks in the ECD spectrum to lower wavenumbers compared to those in the pristine film. Conversely, the peak at 1546 cm^–1^, present in the PEDOT:PSS film due to the DEG additive, is not observed in the ECD spectrum. While the majority of peaks in the ECD spectrum are similar to those in the pristine film, the notable differences highlighted earlier indicate significant structural changes. These differences suggest that PEDOT:PSS undergoes modifications even before its integration into the device. Initially, comparing the Raman spectrum of our PEDOT:PSS film with the literature revealed distinct differences, likely due to the presence of DEG in the solution, suggesting alterations in the film’s structural properties. When this already modified PEDOT:PSS is subsequently incorporated into the ECD, its Raman spectrum shows further modifications. These additional changes are likely induced by interactions with other layers in the ECD, such as ZnO and Al, which could reflect further alterations in the structural environment of the PEDOT:PSS film. These changes might affect its electrical and optical properties and improve charge carrier mobility within the device.^36,42,43,46^

**Figure 3:**
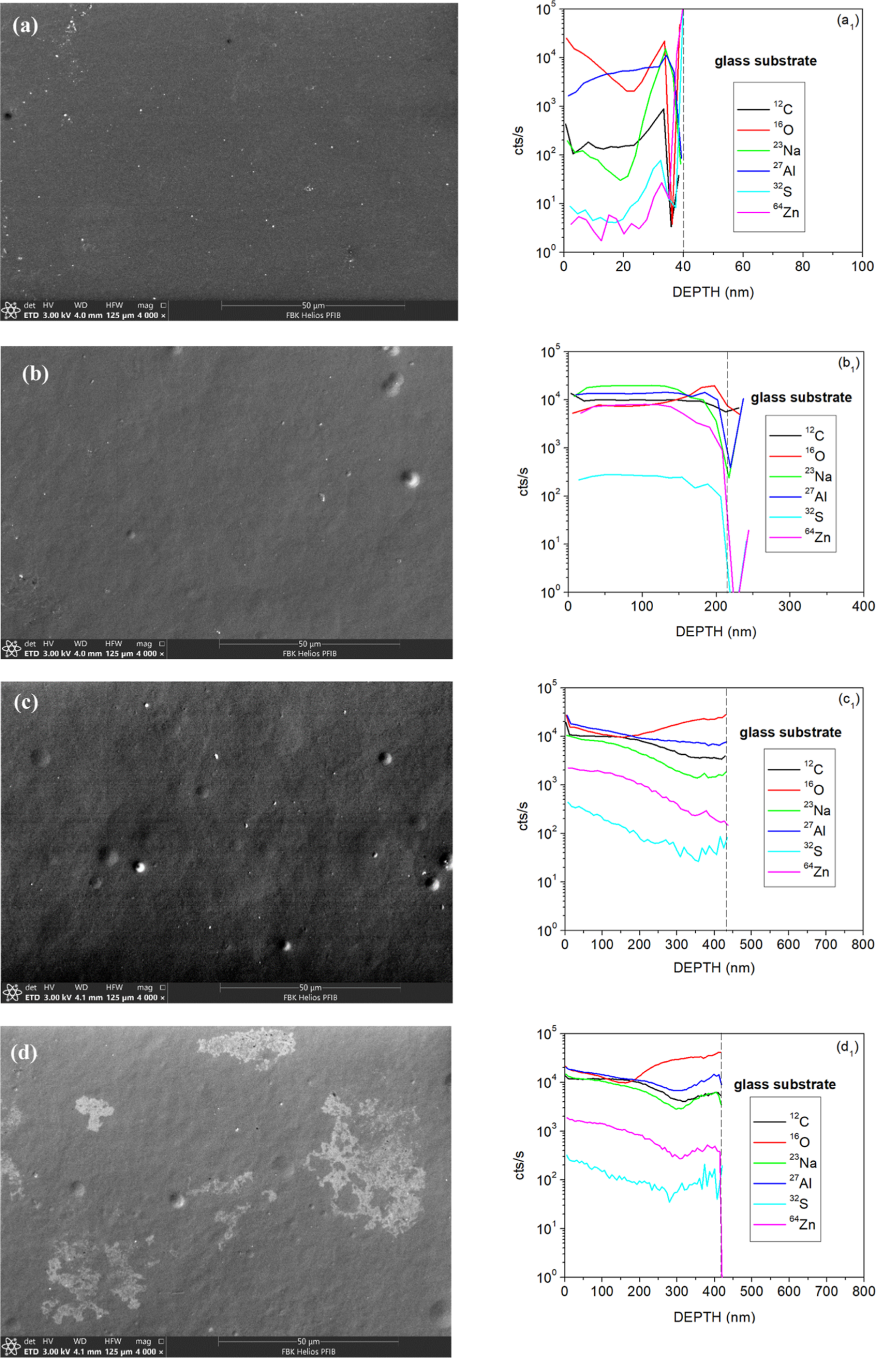
SEM images and corresponding SIMS analyses of (a,a_1_) Al and (b,b_1_) PEDOT:PSS films (deposited on glass substrate), and ECD (c,c_1_) before and (d,d_1_) after cycling process, respectively. The vertical dashed line in SIMS results indicates the interface with the glass substrate.

The SEM and SIMS analyses were conducted on the Al and PEDOT:PSS films deposited on glass substrates via the spray coating technique, as well as on the ECD before and after the cycling process (Figure 3). The SEM image of the Al film (Figure 3a) reveals an almost uniform surface, without significant roughness. The SIMS analysis of this film (Figure 3a_1_) reports the count rates for the secondary ions of carbon (C), oxygen (O), sodium (Na), aluminum (Al), sulfur (S), and zinc (Zn). The oxygen profile suggests the presence of aluminum oxide on the Al film surface. While sulfur is commonly associated with PEDOT:PSS due to its composition, the presence of other elements in the SIMS analysis may be attributed to residual impurities in the glass substrate or contamination during the deposition process. The Al layer thickness, determined by profilometer scans of the SIMS sputtered crater, is approximately 40 ± 2 nm.

In contrast, the SEM image of the PEDOT:PSS film deposited on a glass substrate (Figure 3b) shows a pronounced rough surface compared to the Al layer. Furthermore, the presence of rounded spots in the SEM image, resembling droplets or clusters, indicates irregularities during the spray coating process, leading to excessive deposition of PEDOT:PSS in localized areas. The SIMS analysis of this film (Figure 3b_1_) revealed the unexpected presence of several elements. Particularly noteworthy is the unexpected ion counts on the Al signal, given that only our pristine PEDOT:PSS solution was deposited on the glass substrate. Therefore, the Al signal can be attributed to mass interferences, such as ^27^Al=^12^C_2_H_3_. Additionally, the presence of other elements, such as Na suggests potential contamination within the PEDOT:PSS composition. After the SIMS analysis, the film thickness (244 ± 78 nm) was measured via profilometer scans by scratching the film layer in order to expose the glass substrate.

Considering the surface characteristics observed in both the aluminum and PEDOT:PSS films, it is expected that the roughness of the stacked layers increases, as evidenced in the SEM analysis of the ECD in its initial state (Figure 3c). This increase in roughness poses challenges in accurately identifying individual layers in the device. In the SIMS analysis, high count rates of Al and Na are detected in the Al/ZnO_nano_/PEDOT:PSS film (Figure 3c_1_), which can be attributed to mass interferences with C_2_H_3_ and sample contaminations, respectively. After successive switching, SEM analysis (Figure 3d) reveals visible disruptions in the Al/ZnO_nano_/PEDOT:PSS film compared to its initial state, indicating potential damage or oxidation following repeated cycles. However, SIMS analysis (Figure 3d_1_) shows no substantial differences in elemental compositions and counts between the initial and cycled states, suggesting the device maintains a robust chemical composition. The overall thickness of the ECD layers, excluding the glass substrate, measures approximately 428 ± 128 nm.

### 3.2 Electrochemical and Optical Characterization of ZnO-Based ECD

#### 3.2.1 Electrochemical Characterization

**Figure 4:**
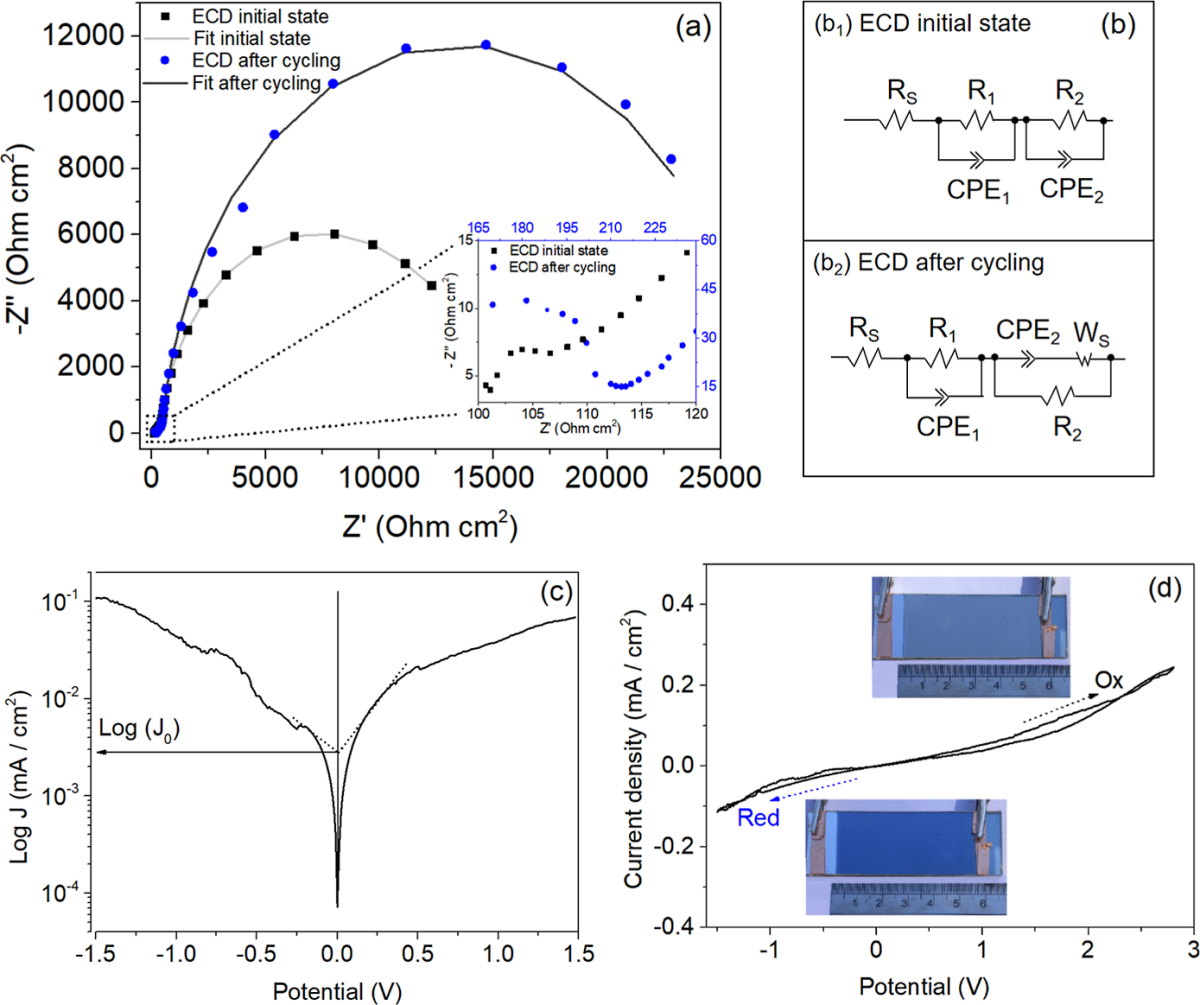
(a) Nyquist plots of the EIS (symbols) from the Al/ZnO_nano_/PEDOT:PSS film and their corresponding fits (lines) before (black squares and light gray line) and after (blue circle and dark gray line) cycling within the voltage range of −1.2 V to +2.8 V (inset: magnification of the high-frequency region of the curves) and (b) equivalent circuit models used to fit the measured EIS data of the ECD before (b_1_) and after (b_2_) cycling. (c) Tafel polarization curve. (d) Electrochemical behavior of the ECD in its initial state within the voltage range of −1.5 V to +2.8 V (insets show visual color changes of the ECD during the oxidation and reduction processes).

To evaluate the electrocatalytic activity during the electrochemical process in the EC device, electrochemical impedance spectroscopy (EIS), Tafel polarization, and cyclic voltammetry (CV) measurements were performed. Figure 4a illustrates the Nyquist plots of the EC device, revealing two consecutive semicircular arcs across the frequency range for both the initial and cycled states of the Al/ZnO_nano_/PEDOT:PSS film. These semicircles denote the impedance behavior of the device and provide insights into its electrochemical properties.

The impedance of the film exhibits noticeable variations in both high and low-frequency regions before and after the switching process. In the ECD in its initial state, the impedance undergoes a significant shift, ranging from relatively low impedance (*Z̀*), below 13 kΩ, to significantly higher values, exceeding 22 kΩ, after 167 cycles of operation. Moreover, the high-frequency arc of the ECD after cycling is notably larger than that observed in its initial state, as shown in the enlarged plot in Figure 4a. This semicircle at high frequencies characterizes the charge transfer resistance between the ZnO_nano_/PEDOT:PSS film (active layer) and the CE interface.

Generally, a larger semicircle in the Nyquist plot indicates higher charge transfer resistance,^47^ which implies that the CE’s electrocatalytic capability for the reduction of redox pairs decrease over time. Furthermore, the inset in Figure 4a reveals an inclined line at the beginning of the low-frequency region, characterized by a steep slope, particularly noticeable in the film after the cycling process. This line is commonly associated with Warburg impedance,^48^ which reflects ion diffusion processes within the device.^11,49−51^ Although the increase in resistance observed in the Nyquist plot may indicate some degradation in the electrochemical properties, the presence of Warburg impedance suggests that ion diffusion is still occurring effectively in the ZnO-based ECD.^50,51^ This demonstrates that the device remains operational and retains its fundamental functional characteristics, even after multiple switching cycles.

The electrochemical parameters were calculated by fitting impedance data using the EIS Spectrum Analyzer software. The equivalent circuit models used to fit the experimental EIS data are shown in Figure 4b. For the ECD in its initial state, the equivalent circuit model (Figure 4b_1_) consists of a series connection of a resistance (*R*_s_) with a parallel connection of a high-frequency resistance (*R*_1_) and a high-frequency constant phase element (CPE_1_). This is followed by a series connection of a low-frequency resistance (*R*_2_) in parallel connection with a low-frequency constant phase element (CPE_2_).

Given the variations in the EIS data, a distinct equivalent circuit model (Figure 4b_2_) was used to fit the experimental EIS data of the ECD after the cycling process, reflecting the differences in the film’s behavior in its initial state and after 167 operational cycles. This model comprises a series connection of a resistance (*R*_s_) with a parallel connection of a high-frequency resistance (*R*_1_) and a high-frequency constant phase element (CPE_1_). This is followed by a series connection of a low-frequency constant phase element (CPE_2_) and a Warburg impedance (*W*_s_), both in series, which are in parallel connection with a low-frequency resistance (*R*_2_). The resulting fits from these equivalent circuit models are shown in Figure 4a and are in good agreement with the experimental EIS curves.

In these circuits, *R*_s_ represents the series resistance related to the sheet resistance of the Al film and electrical connections, *R*_1_ is related to the high-frequency region and represents the charge transfer resistance between the active layer and the counter electrode interface, *R*_2_ is the reaction resistance associated with the reaction kinetics in the low-frequency region and depends on the applied overpotential, *W*_s_ denotes the Warburg diffusion impedance of the electroactive species, while CPE represents a constant phase element that describes the double-layer capacitance of the active layer.^52,53^ These parameters are summarized in Table 1, which confirms a notable increase in charge transfer resistance (*R*_1_) after 167 continuous cycles of oxidation/reduction reactions.

**Table 1:** Results of Fitted EIS Data for the Al/ZnO_nano_/PEDOT:PSS Film in Its Initial State (1st Cycle) and after 167 Operational Cycles in the Voltage Range of −1.2 V to +2.8 V

Sample	*R*_s_ (Ω)		*R*_1_ (Ω)		*R*_2_ (Ω)		*W*_s_ (Ω s^–0.5^)	
	first	167th	first	167th	first	167th	first	167th
ZnO-based ECD	102.21 ± 1.73	87.68 ± 3.51	459.70 ± 7.19	26,081 ± 3.96	13,477 ± 4.10	406.16 ± 3.81		1.67 × 10^–5^ ± 4.38

Figure 4c shows the Tafel plot for the EC device in its initial state, which exhibits a nearly symmetrical shape.^52^ The area at the middle potential with a defined slope corresponds to the Tafel polarization zone.^54^ As shown in Figure 4c, the current density (*J*) of the cathode branch line segment epitaxy to the potential of 0 V is defined as the exchange current density (*J*_0_), which varies inversely with the charge transfer resistance (*R*_1_) value.^55^ It can be observed from Figure 4c that *J*_0_ for the EC device shows a small slope, indicating a low exchange current density (*J*_0_) and, consequently, a high charge transfer resistance. This result suggests that the Tafel polarization measurement is consistent with our EIS data.

In Figure 4d, we present the cyclic voltammogram (CV) assessing the redox response of our electrochromic device in its initial state. The operating voltage was controlled between −1.5 V to +2.8 V at a scan rate of 100 mV.s^–1^. The voltammogram displays two detectable reduction and oxidation regions, indicative of the presence of a redox couple within the system. Notably, the CV exhibits an asymmetrical geometry between these regions, revealing a nonsymmetric behavior during the oxidation and reduction processes in the device, indicating a potentially low reversibility of the redox couple.^56,57^

Specifically, the CV analysis indicates that PEDOT:PSS film undergoes a reduction reaction when a negative voltage is applied to the WE (PEDOT:PSS), resulting in color change in the ECD. Conversely, applying a positive voltage oxidizes the PEDOT:PSS film, causing the ECD to bleach. Therefore, as depicted in the photographs (insets in Figure 4d), the color of our ZnO-based ECD changes reversibly from light blue (bleached state) to dark blue (colored state) with applied voltages of −1.5 V and +2.8 V, respectively. This reversible redox process within the PEDOT:PSS layer facilitates its electrochromic behavior, enabling the color-switching phenomenon through the insertion and extraction of electrons (e^–^) and ions (M^+^) into and out of the film.^58−60^

### 3.3 Optical Properties of the Electrochromic Device

**Figure 5:**
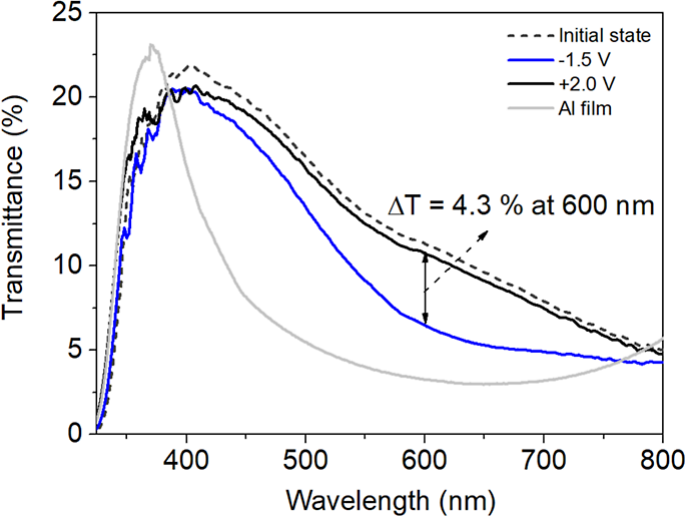
Optical transmittance spectra of the Al film (gray line) and the ECD in its initial (dashed line), bleached (black line), and colored (blue line) states measured at applied voltages of −1.5 V for *T*_c_ and +2.0 V for *T*_b_.

Figure 5 shows the optical transmittance of the ECD containing the glass/Al/ZnO_nano_/PEDOT:PSS structure with an active area of 5.5 × 2.5 cm^2^ in the initial, colored, and bleached states. In addition, the figure includes the spectral transmittance of the Al film counter electrode before the deposition of ZnO and PEDOT:PSS layers. The transmittance spectra of the ECD in colored and bleached states were recorded at −1.5 V and +2.0 V, respectively. These measurements reveal differences in device transmittance between its bleached and colored states in the visible–NIR spectrum (∼410–800 nm). The transmittance modulation (Δ*T*) was calculated using the equation Δ*T* = *T*_b_ – *T*_c_, where T_b_ and *T*_c_ represent the transmittances of the bleached and colored states, respectively. Notably, the most significant Δ*T* value was observed at the wavelength of 600 nm. The transmittance spectrum measured during oxidation indicates that the ECD did not fully return to its original color, suggesting that a voltage higher than +2.0 V would be necessary to restore its initial transmittance state. This observation underlines the importance of optimizing the applied voltage parameters to enhance the electrochromic device’s performance across its operating range.

**Figure 6:**
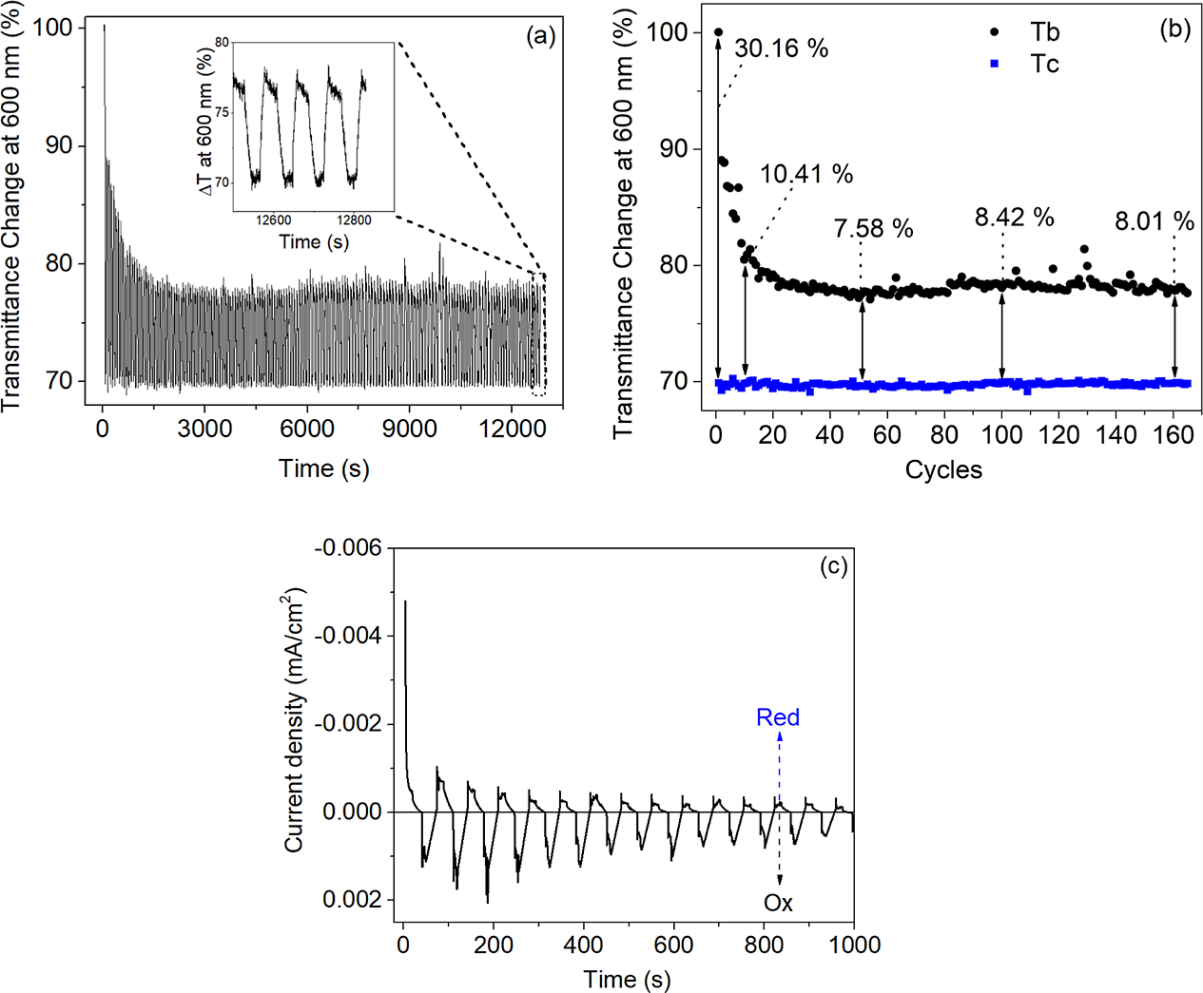
(a) Cycling performance of the ZnO-based ECD (at 600 nm) during successive potential switches between −1.2 V (15 s) and +2.8 V (10 s), (inset: magnification of the final cycles). (b) Transmittance modulation (Δ*T*) as a function of the number of operation cycles. (c) Chronoamperogram of the ECD under the same cyclic switching conditions.

To evaluate the electrochromic performance of the ECD, an in situ kinetic study was conducted by monitoring the relative transmittance changes (Δ*T*) during chronoamperometry cycling (Figure 6a,b). The initial coloration and bleaching potentials were set at −1.5 V and +2.0 V, respectively, as shown in Figure 5, to evaluate the ECD’s optical transmittance as a function of these applied potentials. After analyzing the data, it was observed that −1.5 V effectively achieved coloration, while +2.0 V did not fully restore the ECD’s color to its initial state. Consequently, the potentials to be applied to the ECD during the cycling process were adjusted to −1.2 V and +2.8 V. This adjustment is intended to optimize performance and minimize degradation by using a narrower voltage window for coloration and ensuring more effective bleaching with a higher voltage.

Figure 6a shows the in situ transmittance changes of the ECD at 600 nm over a preset period of approximately 12,800 s (∼4 h). During this period, continuous potentials of −1.2 V for 15 s (followed by LSV for 25 s, ranging from −1 V to 0 V) and +2.8 V for 10 s (followed by LSV for 25 s, ranging from +1 V to 0 V) were applied to the ECD, resulting in a total of 167 cycles (Figure 6b). This in situ transmittance analysis reveals significant variations in Δ*T* of the ECD in response to the applied potentials, particularly during the first 10 cycles of the measurement (Figure 6a,b). As shown in Figure 6b, Δ*T* initially reached 30.16% in the first cycle, then decreased progressively, dropping to 10.41% by the 10th cycle. Following this initial decrease, Δ*T* stabilized around an average value of 8.5 ± 0.7% for the remaining cycles. These significant variations in Δ*T* align with previous studies,^61,62^ which suggest that the prominent color change observed during the initial cycles is primarily due to the coloration of the polymeric material (PEDOT:PSS) and may not fully represent the overall device performance. As the cycling progresses, the influence of the ZnO-based electrochromic device becomes more evident, with the transmittance change stabilizing at values that better reflect the device’s actual performance. This transition from an initially polymer-dominated coloration to a more stable state reflects the evolving role of the electrochromic material over time, leading to a more accurate representation of the ECD’s true behavior.

Just as Δ*T* exhibited significant variations during the initial cycles, the current density also oscillated considerably at the beginning of the cycling process, as shown in Figure 6c. Following these initial cycles, both current density and Δ*T* appear to reach a plateau, suggesting a possible stabilization in both the charge dynamics required for the device’s color change and optical modulation. After these initial fluctuations, the Δ*T* value more closely aligns with the variation observed in the optical transmittance spectra shown in Figure 5.

The observation that Δ*T* decreased from 10.41% to 8.01% during the cycling process (excluding the first 10 cycles) suggests a slight degradation in the device’s performance, potentially influenced by contaminants identified in the SIMS analysis. However, the stabilization of Δ*T* around 8.5 ± 0.7% after the initial fluctuations, combined with a consistent current density, indicates that the ECD maintains stable performance over time. This stability is further supported by the device’s continuous operation for nearly 4 h. A zoomed-in section of the last cycles in Figure 6a demonstrate that the device did not fail or degrade but rather that the measurement period was terminated due to the UV–vis spectrophotometer’s operational limits. The stability at the end of the measurement suggests that the device would likely have continued to maintain its performance if the cycling process had been extended. Furthermore, the relatively long duration of each cycle, due to the applied coloration and bleaching potentials intercalated with LSV, likely contributed to the ECD’s stability despite the relatively modest number of cycles achieved (Figure 6b). This procedure may have also contributed to maintaining the device’s good performance and durability. Thus, the ZnO-based ECD demonstrated the capability to maintain reliable operation for at least 12,800 s.

**Figure 7:**
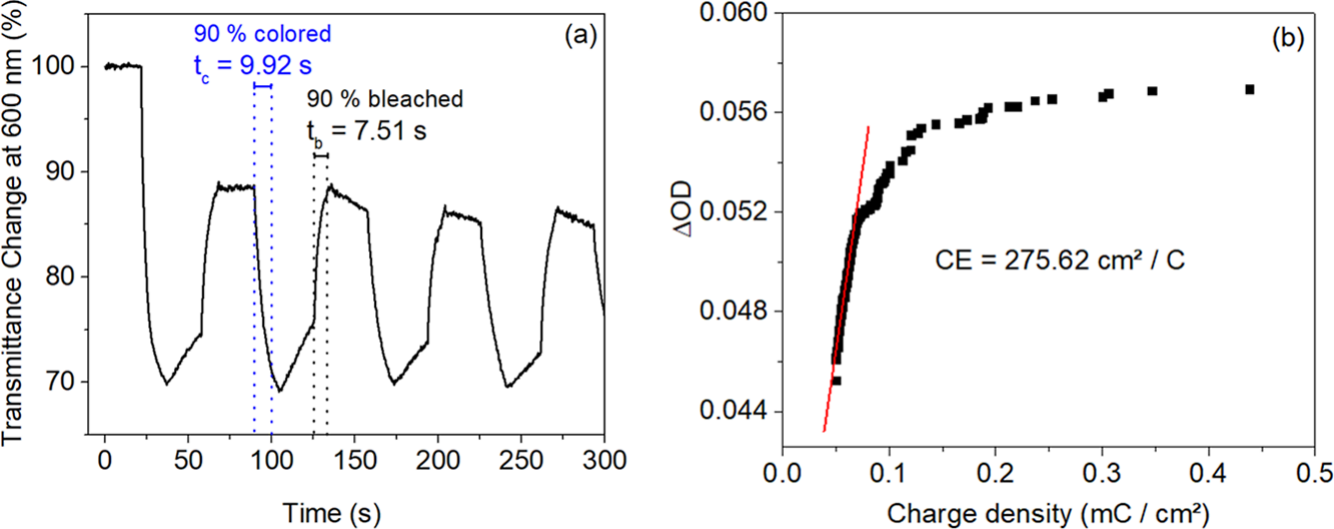
(a) Switching times between the bleached and colored states extracted from the cycling process. (b) Optical density change (ΔOD) as a function of the injected charge density; the coloration efficiency (CE) is estimated from the fitted slope of the linear region of the plot.

The response time from the transition to the colored state (*t*_c_) to the bleached state (*t*_b_) is an important aspect of the EC system, defined as the time required for the device to achieve 90% of its fully colored or bleached state.^57^ Extracted from the in situ optical transmittance spectrum (Figure 6a), the response times for the colored and bleached states were estimated at 9.92 s and 7.51 s, respectively, as shown in Figure 7a. Compared to other PEDOT:PSS-based devices,^57,63^ the response times are shorter, indicating that our ECD exhibits a relatively fast color-switching speed. Additionally, when compared to our previously manufactured Alq_3_-based ECD,^31^ the coloring time showed a slight increase, while the bleaching time was significantly reduced. Moreover, our current ZnO-based ECD maintained optical contrast and stability for a significantly longer period than the 4000 s evaluated in that previous study.^31^

Coloration efficiency (CE) is another important measure for assessing the electrochromic performance of EC materials. CE quantifies the change in optical density (OD) per unit of inserted charge density (*Q*/*A*, where *A* is the area and *Q* is the charge) during the coloration process. CE can be determined using the following equation^64^
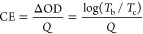
In this equation, *T*_b_ and *T*_c_ correspond to the transmittances in the bleached and colored states, respectively, at a specific wavelength. Since coloration efficiency can be influenced by the coloring of the polymeric material, which is typically more pronounced during the initial cycles, CE was calculated in relation to Δ*T* throughout the entire cycling process (excluding the first cycle), rather than for a single full switch. Figure 7b displays ΔOD at a wavelength of 600 nm as a function of the charge inserted into the ECD under a potential of −1.2 V. From this figure, the CE value is estimated by the slope of the ΔOD – *Q* plot when *Q* is close to 0. According to the calculation of the slope, the CE value of the ECD is 275.62 cm^2^/C. This value not only aligns with similar ECDs^65−68^ but also surpasses other PEDOT:PSS-based devices,^63,69−72^ including our previously developed Alq_3_-based ECD.^31^ This remarkable result underscores the energy-efficient nature of our ZnO-based ECD, paving the way for its potential integration into a wide range of sustainable technologies.

## 4 Conclusions

This study successfully demonstrated the fabrication of an all-solid-state electrochromic device, featuring a simplified glass/Al/ZnO_nano_/PEDOT:PSS architecture, achieved using a simple and cost-effective spray coating technique. Notable changes in transmittance modulation were observed during the initial cycles, primarily attributed to the coloring of the film containing the electrochromic material, resulting in an increase in optical contrast that does not accurately reflect the device’s performance. Subsequently, the Δ*T* exhibited lower values, probably indicating the real performance of the device, which stabilized at around 8.5 ± 0.7% for the remaining 157 cycles of continuous switching between −1.2 V and +2.8 V. Furthermore, an analysis of the cycling performance and coloration efficiency of the ZnO-based ECD highlighted its relatively fast electrochromic responses and excellent coloration efficiency, with a notable CE value of 275.62 cm^2^/C. This high CE also confirms the device’s long-term electrochemical stability and highlights its potential for practical applications, including security features, displays, and energy-efficient technologies. Future research could focus on optimizing the preparation methods for ZnO nanorods, PEDOT:PSS, and Al layers, aiming to improve deposition uniformity and determine the optimal thickness of each layer. Additionally, reducing charge transfer resistance during the cycling process could further enhance both performance and stability. These improvements would broaden the device’s applicability across various optical and electronic technologies.
